# Mapping and candidate gene screening of tomato *Cladosporium fulvum-*resistant gene *Cf-19*, based on high-throughput sequencing technology

**DOI:** 10.1186/s12870-016-0737-0

**Published:** 2016-02-25

**Authors:** Tingting Zhao, Jingbin Jiang, Guan Liu, Shanshan He, He Zhang, Xiuling Chen, Jingfu Li, Xiangyang Xu

**Affiliations:** College of Horticulture, Northeast Agricultural University, Harbin, 150030 China

**Keywords:** *Cf-19* gene, *Cf-4/9* locus, *Solanum lycopersicum*, *Cladosporium fulvum*, Tomato leaf mold, SLAF-seq

## Abstract

**Background:**

Tomato leaf mold is a common disease in tomato cultivation. This disease is caused by *Cladosporium fulvum*, which has many physiological races and differentiates rapidly. *Cf* genes confer resistance to *C. fulvum*, and the *C. fulvum*-tomato pathosystem is a model for the study of gene-for-gene interactions. Plants carrying the *Cf-19* gene show effective resistance to *C. fulvum* in the field, and can be used in breeding and resistance mechanism studies as new resistant materials. In this study, we used F_2_ bulk specific-locus amplified fragment sequencing (SLAF-seq) and parental resequencing methods to locate and characterize the *Cf-19* gene.

**Results:**

A total of 4108 Diff_markers and three association regions were found in association analysis. A 2.14-Mb region containing seven *Cf*-type genes was identified in further analysis based on data from SLAF-seq and parental resequencing. Two candidate genes, Solyc01g006550.2.1 and Solyc01g005870.1.1, were screened out by quantitative real-time PCR (qRT-PCR) analysis. Sequence analysis showed that Solyc01g006550.2.1 (an allelic locus of *Cf-0*) in CGN18423 was a novel homologue of the *Cladosporium* resistance gene *Cf-9* (*Hcr9s*) in the *Cf-4/9* locus. The marker P7, which cosegregated with the resistant trait, was developed based on sequence mutation of the Solyc01g006550.2.1 locus in CGN18423.

**Conclusions:**

The *Cf-19* gene was mapped to the short arm of chromosome 1. The candidate genes Solyc01g006550.2.1 and Solyc01g005870.1.1 showed related amino acid sequence structures and expression patterns. Solyc01g006550.2.1 had a close evolutionary relationship with the functional *Hcr9* members *Cf-4* and *Cf-9*, and was very different from non-functional members. The results from this study will facilitate the breeding of cultivars carrying the *Cf-19* gene and provide a basis for further gene cloning, resistance gene evolution and plant resistance mechanism studies.

**Electronic supplementary material:**

The online version of this article (doi:10.1186/s12870-016-0737-0) contains supplementary material, which is available to authorized users.

## Background

Tomato leaf mold disease, caused by the biotrophic fungus *Cladosporium fulvum*, is a serious disease of *Solanum lycopersicum* (tomato) [1]. This disease can reduce both fruit yield and quality, and sometimes even kill tomato plants. In compatible interactions with susceptible tomato plants, fungal spores germinate on the abaxial surface of leaves and enter the leaf apoplast through stomata. Hyphae emerge through the stomata and continue to grow and ramify. Finally, the infected cells undergo necrosis [2]. Tomato *Cf* genes confer resistance to *C. fulvum* and mediate incompatible interactions between *C. fulvum* and tomato plants. In incompatible interactions, fungal hyphae are arrested in their development soon after penetration of the sub-stomatal cavity, and their growth is restricted to limited necrotic lesions [2]. Plant breeders introduced *Cf* resistance genes from wild *Solanum* species into cultivated tomato to control the disease many years ago [3, 4], and this is still an efficient method in tomato cultivation today. A number of *Cf* genes have been introgressed for use in commercial breeding, but this artificial selection has created evolutionary pressure on *C. fulvum*. To date, many *Cf* genes have been overcome by *C. fulvum* carrying matching avirulence genes (*Avr*). A race that has evolved to overcome the resistance genes *Cf-2*, *Cf-4*, *Cf-5*, *Cf-9*, and *Cf-11* was reported by Lindhout et al. [5]. In northeast China, the *C. fulvum* physiological races 1.2.3.4 and 1.2.4 have evolved to overcome the *Cf-4* gene and become the dominant races in only nine years [6]. The number of *Cf* genes that can be used in breeding is decreasing, making it necessary to identify new resistance genes and breed them into tomato cultivars.

*Cf* genes, which encode predicted membrane-bound proteins with extracytoplasmic leucine-rich repeats (LRRs) [7], confer resistance to specific races of *C. fulvum* through recognition of *Avr* peptides secreted into the leaf apoplast during infection [8]. In the tomato–*C. fulvum* interaction, a strict correlation exists between the triggering of a hypersensitive response (HR) and resistance, as the various Avrs induce a specific HR in tomato genotypes carrying a matching *Cf* resistance gene [9]. In 1980, 24 *Cf* genes located on 12 chromosomes were reported in tomato [10, 11]. Later studies showed that the *Cf* genes were organized into clusters of resistance gene homologues, which have been designated *Hcr2*s and *Hcr9*s for homologues of the *Cladosporium* resistance genes *Cf-2* and *Cf-9*, respectively [12]. *Hcr2* loci, including the *Cf-2* and *Cf-5* loci, have been mapped to the short arm of chromosome 6 [13]. Three *Hcr2* homologues were found at the *Cf-2* locus. The *Cf-2.1* and *Cf-2.2* genes are nearly identical, and are functional genes that confer resistance to strains of *C. fulvum* that carry the matching *Avr2* gene. The other homologue (*Hcr2-2A*) is not functional [14]. At the *Cf-5* locus, four homologues were found, among which *Hcr2-5C* is the corresponding functional gene *Cf-5* [15]. The *Cf-4* and *Cf-9* loci, each comprising five *Hcr9*s, have been mapped to the short arm of chromosome 1 [16]. *Hcr9-4D* and *Hcr9-9C* are the functional genes *Cf-4* and *Cf-9*, respectively [7, 17]. Tomato plants carrying the *Cf-19* gene have shown efficient resistance in the field and no infection has been reported for this gene to date. *Cf-19* was assigned to the long arm of chromosome 2 in 1980 [11]. Our previously study showed that *Cf-19* was a dominant gene [18] and induced a remarkable HR in tomato plants inoculated with *C. fulvum* physiological race 1.2.3.4, indicating that it is a functional member of the *Cf* gene family.

SLAF-seq, which is based on high-throughput sequencing, is a recently developed high-resolution strategy for large-scale *de novo* single nucleotide polymorphism (SNP) discovery and genotyping [19]. This method is relatively low-cost and efficient, and can be used for gene and quantitative trait locus (QTL) mapping. The efficiency of this approach was tested in rice and soybean, and it was successfully used to construct genetic maps for sesame (*Sesamum indicum* L.) [20]. We applied this approach to an F_2_ population in combination with genome resequencing of the parents to map and characterize the *Cf-19* gene rapidly.

## Results

### Disease severity ratings and genetic analysis of the *Cf-19* gene

CGN18423 and F_1_ plants were resistant to the *C. fulvum* race 1.2.3.4, while Moneymaker plants were susceptible. Chi-square analysis showed that the segregation ratio of the resistant and susceptible individuals of the F_2_ population was 3:1. Resistant and susceptible BC_1_ plants segregated according to the expected ratio of 1:1 (Table 1).

**Table 1:** Genetic analysis of *Cf*-19 disease resistance in different generations

Generation		No. of plants		Expected segregation ratio (R:S)	χ^2^
	Total	Resistant (R)	Susceptible (S)		
CGN18423	30	30	0		
Moneymaker	30	0	30		
F_1_	30	30	0		
F_2_	469	339	130	3:1	1.70
BC_1_	102	45	57	1:1	1.19

χ^2^_0.05, 1_ = 3.84

### High-throughput sequencing analysis

**Fig. 1:**
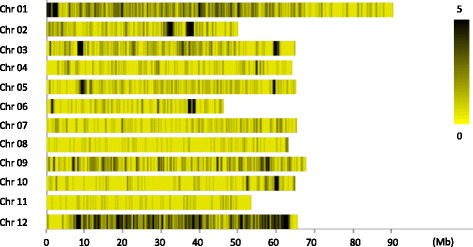
Marker distribution on the chromosomes. Abscissa: position of SLAF tags on the chromosomes; ordinate: chromosome ID. The darker the color, the more SLAF tags were present

A total of 20.15 Gb data including 99.75 M reads and 4.07 Gb data including 20.22 M reads were obtained from parental genome sequencing and F_2_ bulk SLAF-seq, respectively (Table 2). From the 119,504 SLAF tags, 4108 Diff-markers were obtained. A distribution diagram of the markers on each chromosome was drawn according to the results of SLAF positioning on the genome (Fig. 1).

**Table 2:** Sequencing data of each sample

Sample	Sample-ID	Read length	Total reads	Total nucleotides	GC (%) percentage
Resistant pool	aa	101 + 101	11,033,299	2,222,039,840	35.39 %
Susceptible pool	ab	101 + 101	9,184,513	1,851,224,404	35.36 %
CGN18423	M	101 + 101	49,250,898	9,948,681,396	36.96 %
Moneymaker	P	101 + 101	50,497,727	10,200,540,854	37.41 %

### Association analysis and candidate gene screening

**Fig. 2:**
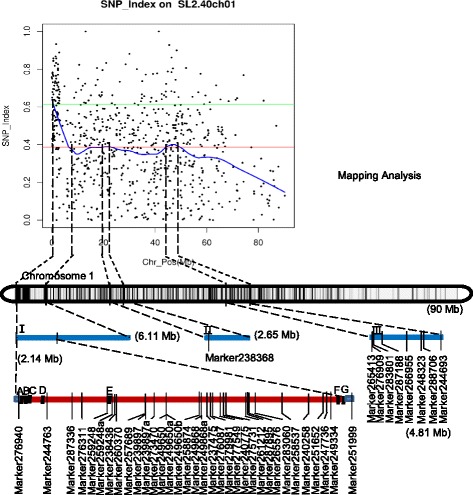
Genetic and physical maps of mapping regions and the mapping analysis process. Three association regions are shown as I, II and III based on the F_2_ SLAF-seq analysis. These regions are 6.11, 2.65 and 4.81 Mb in size, respectively. Forty-three markers distributed in the three regions and seven candidate genes (A-G) in a 2.14-Mb region were screened out by the combination of F_2_ SLAF-seq and parental resequencing. A, Solyc01g005730.2.1; B, Solyc01g005760.2.1; C, Solyc01g005780.1.1; D, Solyc01g005870.1.1; E, Solyc01g006550.2.1; F, Solyc01g008390.1.1; G, Solyc01g008410.1.1

According to the results of ΔSNP index calculation, all Diff-Markers were distributed on chromosome 1. Regions with three or more consecutive Diff-markers were regarded as association regions. We found three association regions including 203 Diff-markers and 244 genes on chromosome 1 in SLAF-seq analysis (Table 3). To further narrow the mapping region, SNPs in the association regions from parental resequencing were analyzed in combination with the F_2_ bulk SLAF-seq data. Forty-three SNPs showed the Moneymaker base type in the susceptible pool, and both parental base types in the resistant pool were screened out. Of the 43 SNPs, 34 were distributed in an approximately 2.14-Mb region in association region I, one was in association region II and eight were in association region III (Additional file 1: Table S1). Further analysis was carried out based on the results of association analysis and gene function annotation. Finally, seven genes with *Cf*-type characters were screened out, all of which were inside the 2.14-Mb association region I that was identified in the previous SNP analysis. These genes were Solyc01g005730.2.1, Solyc01g005760.2.1, Solyc01g005780.1.1, Solyc01g005870.1.1, Solyc01g006550.2.1, Solyc01g008390.1.1 and Solyc01g008410.1.1, respectively (Fig. 2).

**Table 3:** Detail information of association regions

Chromosome ID	Start (genome positon)	End (genome positon)	Size (Mb)	Diff_Marker number	Gene number
@SL2.40ch01	10,814	6,124,581	6.11	118	236
@SL2.40ch01	19,985,817	22,639,492	2.65	23	2
@SL2.40ch01	44,283,583	49,095,823	4.81	62	6

### Quantitative real-time PCR analysis

**Fig. 3:**
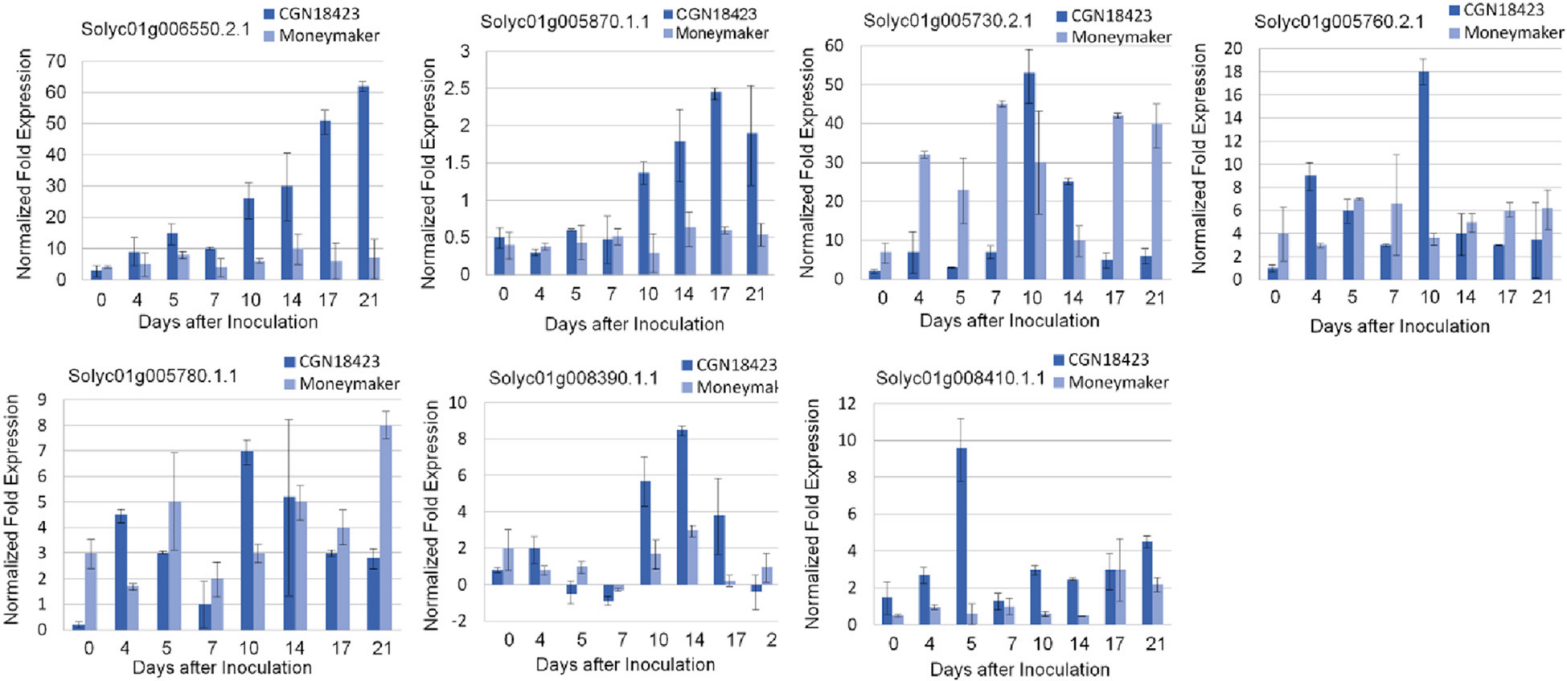
qRT-PCR analysis of the relative gene expression of seven candidate genes

According to the results of qRT-PCR analysis, of the seven candidate genes, two (Solyc01g005870.1.1 and Solyc01g006550.2.1) showed expression patterns related to the resistance response process. As shown in Fig. 3, the Solyc01g006550.2.1 gene was expressed at a low level before inoculation, and increased slightly after inoculation. This expression level was maintained for about 5 days, and then increased rapidly at 7 days after inoculation (DAI) and kept increasing during the following days. The highest expression was at 21 DAI, which was 62-fold higher than the 0 DAI value. The expression level of the Solyc01g005870.1.1 gene was relatively lower at every stage compared with the corresponding level of Solyc01g006550.2.1, while the general expression patterns of the two genes were similar. All other five genes showed unrelated expression patterns during the whole penetration process.

### Candidate loci sequencing and sequence analysis

**Fig. 4:**
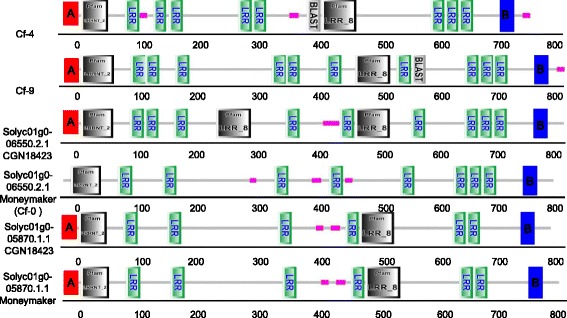
Protein structure comparative analysis of candidate loci and classical Cf proteins. All proteins contain a signal peptide, leucine-rich repeats (LRR) and a transmembrane region except for Cf-0 of Moneymaker, which lacks the signal peptide domain. The Solyc01g006550.2.1 locus proteins in CGN18423 and Moneymaker are very different in domain type and LRR number, while the Solyc01g005870.1.1 locus proteins in CGN18423 and Moneymaker contain the same domains and LRR number. The red box with the letter “A” inside shows the signal peptide domain and the blue box with the letter “B” inside shows the transmembrane region

The Solyc01g005870.1.1 and Solyc01g006550.2.1 loci of CGN18423 and Moneymaker were sequenced successfully (GenBank: KT874515, KT874514, KT845954, KT874513). Sequence alignment showed that the DNA sequences of both loci contained mutations between CGN18423 and Moneymaker. At the Solyc01g005870.1.1 locus, the encoded protein contained a signal peptide, 19 LRRs and a transmembrane region. Several SNPs existed in the coding region, but no difference was found in the conserved domain distribution between CGN18423 and Moneymaker. At the Solyc01g006550.2.1 locus, a 60-bp insertion was found near the N-terminus of the coding region in CGN18423. This insertion changed the ORF, provided a signal peptide for the original Cf-0, and led to an increased number of LRR domains (from 27 to 30), according to conserved domain and feature analysis (Fig. 4).

**Fig. 5:**
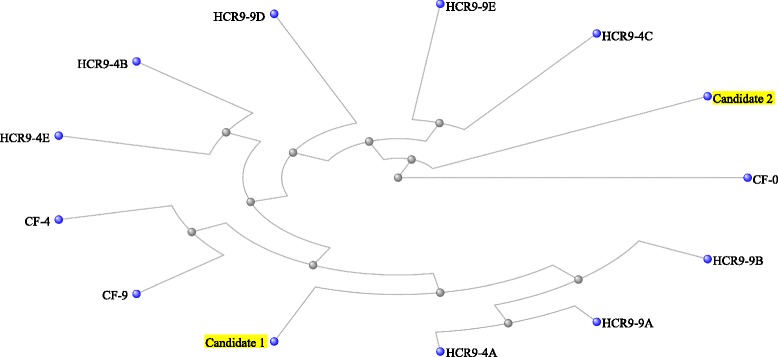
Cluster analysis of candidate and other genes in the *Cf-4/9* locus. Candidate 1 is Solyc01g006550.2.1 of CGN18423 and candidate 2 is Solyc01g005870.1.1 of CGN18423

Multiple DNA sequence alignment of the candidate genes and other *Cf-4/9* locus genes showed that the Solyc01g006550.2.1 locus of CGN18423 had a close evolutionary relationship with the *Cf-4* and *Cf-9* genes. The Solyc01g005870.1.1 locus of CGN18423 showed a short evolutionary distance to the *Cf-0* gene, and the level of sequence divergence was very low (Fig. 5).

### Marker development and linkage analysis

**Fig. 6:**
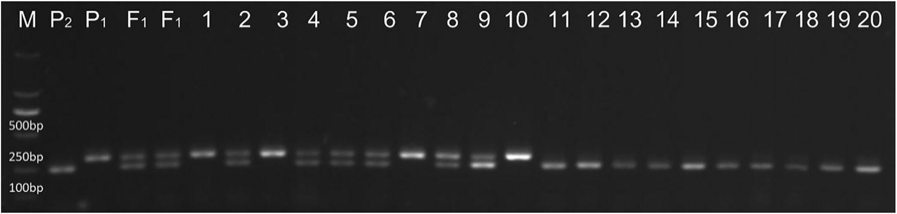
Marker P7 amplification in different generations. P_1_: CGN18423; P_2_: Moneymaker; F_1_: F_1_ plants from the cross of CGN18423 and Moneymaker; 1–10: resistant plants of the F_2_ population; 11–20: susceptible plants of the F_2_ population

A sequence characterized amplified region (SCAR) marker (forward primer: 5’-AGTGCAGAAATGGGTTGTGTA-3’; reverse primer: 5’-CCGGAGATCAAGCTCAACCA-3’) was found to co-segregate with the resistance trait. This marker was developed based on the 60-bp insertion in the Solyc01g006550.2.1 locus in CGN18423. As shown in Fig. 6, a fragment of 300 bp was amplified in CGN18423, a fragment of 240 bp was amplified in Moneymaker, and both of these fragments were amplified in the F_1_ samples. Among 345 F_2_ plants, 72 susceptible plants showed the Moneymaker genotype, 270 resistant plants showed the CGN18423 or F_1_ genotype, and three susceptible plants showed the F_1_ genotype. In the F_3_ lines test, all 131 F_3_-1 plants showed the CGN18423 genotype, the F_3_-2 plants included 42 plants with the CGN18423 genotype, 35 plants with the Moneymaker genotype, and 90 plants with the F_1_ genotype, and all 76 plants of F_3_-3 line showed the Moneymaker genotype. The inoculation and molecular marker identification results of all F_3_ line plants showed consensus.

## Discussion

### The *Cf-19* gene was mapped to the short arm of chromosome 1

*Cf-19* was assigned to the long arm of chromosome 2 in 1980 [11], while this gene was mapped at the short arm of chromosome 1 in this study. Although some Diff_Markers were found on the long arm of chromosome 2 according to the results of F_2_ bulk SLAF-seq analysis, they could not form association regions in further association analysis. Most Diff_Markers were found on the short arm of chromosome 1, and these markers showed us three association regions in association analysis. The position information for these regions was very reasonable for the *Cf* genes were organized into clusters of resistance gene homologues and most *Cf* genes mapped to chromosome 1 or chromosome 6 so far.

Three association regions for the *Cf-19* gene were identified in F_2_ bulk SLAF-seq analysis. This result was not as accurate as we expected. All association regions in this study were on chromosome 1, and this may have been the reason why the location result was not accurate. The arrangements of *Cf* genes on the short arm of chromosome 1 are very complex. Two loci (*Cf-4* and *Cf-9*) of the clustered *Hcr9* genes have been mapped to this region, and each comprises five *Hcr9*s [16]. Additionally, the *Cf-1* gene [21] has been mapped and closely linked to *Cf-4/9* [16]. Other studies have mapped the *Cf-ECP1*, *Cf-ECP2*, *Cf-ECP3*, *Cf-ECP4* and *Cf-ECP5* genes to the short arm of chromosome 1 [22–25]. Many homologous fragments produced by evolutionary events are present in these genes and intergenic regions. These homologous sequences may influence the reads mapping and SNP statistics.

### The candidate gene Solyc01g006550.2.1 of CGN18423 is a new *Hcr9* member in the *Cf-4/9* locus

The *Cf-4* gene originating from *Lycopersicon hirsutum* and the *Cf-9* gene originating from *L. pimpinellifolium* were mapped to the *Cf-4/9* locus [13]. This locus is flanked by conserved lipoxygenase sequences and is very complex, while only a single *Hcr9* gene has been found at the *Cf-4/9* locus in the disease-susceptible cultivar of *Lycopersicon esculentum* Moneymaker [7]; this gene was designated *Cf-0*. The Solyc01g006550.2.1 gene locus in Moneymaker (Solyc01g006550.2.1-Moneymaker) is just the *Cf-0* locus; therefore, our candidate gene Solyc01g006550.2.1 of CGN18423 (Solyc01g006550.2.1-CGN18423) is an allele of *Cf-0*. Sequence analysis showed that a 60-bp insertion was present in the N-terminal coding region, and this insertion resulted in changes in the ORF. Blast search results indicated that no genes were completely homologous to the Solyc01g006550.2.1-CGN18423 gene, which suggests that the candidate gene Solyc01g006550.2.1-CGN18423 is a novel member of the *Cf-4/9* locus.

*Cf* genes encode proteins with classical signal peptide domains in their N-termini, LRRs, and a transmembrane region in their C-termini. *Cf-4* and *Cf-9* have identical C-termini [14], while a significant degree of sequence divergence is found in their N-terminal portions. This difference between *Cf-4* and *Cf-9* produces their recognition specificity [7]. A similar result was found in this study; the C-terminal portion of the candidate gene Solyc01g006550.2.1-CGN18423 had high identity to *Cf-4* and *Cf-9*, while the N-terminal portion contained a high degree of sequence divergence. This result indicates that Solyc01g006550.2.1-CGN18423 may have recognition specificity that has formed based on a similar evolutionary mechanism to *Cf-4* and *Cf-9*. LRRs can form a β-strand/β-turn motif in which the hypervariable residues are solvent-exposed and potentially contribute to recognition specificity. All *Hcr9s* at the *Cf-4/9* locus encode proteins with 27 LRRs with the exception of 4B, which contains 23 LRRs, and *Cf-4*, which comprises 25 LRRs [7]. The candidate gene Solyc01g006550.2.1-CGN18423 encoded 30 LRRs, which is different to all other *Hcr9s*, providing conditions for the formation of a specific motif for ligand recognition. Phylogenetic analysis showed that the candidate gene Solyc01g006550.2.1-CGN18423 had very short evolutionary distances to functional *Hcr9*s including *Cf-4*, *Cf-9*, *Hcr9-9A* and *Hcr9-9B* (adult plants carrying *9A* and *9B* showed a resistance response to *C. fulvum*); these genes were clustered in a branch with a high degree of evolution, and were distinct from other non-functional *Hcr9*s. This result suggested a high possibility that the candidate gene Solyc01g006550.2.1-CGN18423 was our target gene *Cf-19*.

### The marker P7 can be used in marker-assisted selection (MAS) breeding

P7 is a codominant marker that co-segregates with the resistance trait. This marker was designed based on a 60-bp insertion in Solyc01g006550.2.1, making the products in CGN18423 and Moneymaker different in size. This marker was tested in F_2_ plants and different F_3_ lines. Only three F_2_ plants showed an unexpected genotype. These three F_2_ plants were susceptible in the inoculation test, but showed the F_1_ genotype in the P7 test. Hammond-Kosack and Jones [2] suggested that the increased *C. fulvum* invasion of host tissue and the higher titer of intercellular fluid required to elicit a necrotic or chlorotic response in lines where a *Cf* gene was present in a heterozygous state indicates that *Cf* genes are incompletely dominant. The *Cf-19* gene may also be slightly affected by incompletely dominant inheritance, leading to a higher disease severity score for some heterozygous plants, and these plants were divided into the susceptible bulk. Although not all samples gave consistent results in the inoculation and P7 tests, the veracity of P7 in genotype identification is sufficient for MAS breeding work.

### The combination of SLAF-seq and parental resequencing is effective for gene mapping

The SLAF-seq method provides significant advantages for developing large numbers of trait-related markers and target gene mapping. Genome resequencing can provide more details about genome sequences than SLAF-seq, and this information is useful to improve the precision of gene mapping. In this study, three association regions totaling 13.57 Mb in size were obtained in F_2_ bulk SLAF-seq analysis, and a 2.14-Mb region with seven candidate genes was finally identified based on further SNP analysis using data from parental resequencing. The results of qRT-PCR analysis showed the accuracy of the mapping region at the transcriptional level. The current study also indicates that the combination of F_2_ bulk SLAF-seq and parental resequencing is a good choice for both relatively low cost and high efficiency gene mapping and trait-related gene screening.

### Impact of the current work on plant breeding and resistance mechanism research

Breeding to obtain resistant cultivars is an efficient method for the control of leaf mold outbreaks. The differentiation of *C. fulvum* physiological races is very rapid, and new *Cf* resistance genes are of great significance to breeding. *Cf-19* was located on the short arm of chromosome 1 and one candidate gene Solyc01g006550.2.1 was assigned to the *Cf-4/9* locus, while another candidate gene Solyc01g005870.1.1 was mapped close to the *Cf-4/9* locus, which indicates that the *Cf-19* gene may have a direct evolutionary relationship with *Cf-4* and *Cf-9*. The characteristics of *Cf-19* gene introgression to other cultivars may be similar to those of *Cf-4* and *Cf-9* and we have lots of experience in breeding cultivars carrying the *Cf-4* or *Cf-9* gene. Our location results will help guide the breeding of cultivars carrying the *Cf-19* gene. The *Cf* gene family is important for studying plant resistance (*R*) gene evolution and R/Avr interaction mechanisms [26–28]. The candidate gene Solyc01g006550.2.1 is a new member of the *Hcr9*s and has a close evolutionary relationship with *Cf-4* and *Cf-9*. It provides us a new gene material for plant resistance mechanism and R gene evolution research. The results we obtained in the present study were based on genetic mapping, expression pattern analysis and sequence analysis, but to clone the *Cf-19* gene correctly, we still need functional verification such as virus-induced gene silencing (VIGS) and transgenic experiments.

## Conclusions

In this study, we used F_2_ SLAF-seq and parental resequencing to locate the *Cf-19* gene. A total of 4108 Diff-markers were obtained. Three association regions consisting of 203 Diff-markers and 244 genes were found on chromosome 1. A 2.14-Mb region with seven candidate genes on the short arm was obtained through SNP analysis, and the candidate genes Solyc01g006550.2.1 and Solyc01g005870.1.1 were identified from these seven genes by qRT-PCR analysis. The candidate gene Solyc01g006550.2.1 is a new member of the *Hcr9*s in the *Cf-4/9* locus. A SCAR marker (P7) that was co-segregant with the resistance trait was developed that can be used in MAS breeding work. These results provide a basis for *Cf-19* gene cloning and application of the *Cf-19* gene in breeding.

## Methods

### Plant materials and nucleotide extraction

The resistant line *S. lycopersicum* CGN18423 containing the *Cf-19* gene (kindly provided by the Institute of Vegetable and Flowers, Chinese Academy of Agricultural Sciences) was crossed with the susceptible line *S. lycopersicum* Moneymaker. The resulting F_1_ plants were self-crossed and backcrossed with Moneymaker to obtain F_2_ and BC_1_. Three F_2_ plants with different genotypes were self-crossed to obtain F_3_ lines. All plants were grown at the Horticultural Experimental Station of Northeast Agricultural University.

At the five- to six-leaf stage, the seedlings of CGN18423, Moneymaker, F_1_, F_2_, BC_1_ and F_3_ plants were inoculated with *C. fulvum* race 1.2.3.4. The plants were assessed for disease severity at 15 DAI. Inoculation and assessment of disease severity ratings were performed as described by Wang et al. [6]. The disease severity symptoms of the plants were converted to a disease score of 0–9 points—0 points: no visible signs of infection; 1 point: 1-mm-diameter white spots or necrotic spots on the upper sides of leaves; 3 points: 2 to 3-mm-diameter yellow spots on the upper sides of leaves, some white mold on the lower sides of leaves, no spores formed; 5 points: 5 to 8-mm-diameter yellow spots on the upper sides of leaves, abundant white mold on the lower sides of leaves, a few spores formed; 7 points: 5 to 8-mm-diameter yellow spots on the upper sides of leaves, some black mold, many spores on the lower sides of leaves, also some black mold and no spores on the upper sides of leaves; 9 points: masses of spores formed on both sides of the leaves. Plants with a disease score of 0 to 3 points were classified as resistant whereas those with a score of 5 to 9 points were classified as susceptible.

Based on the inoculation results, 50 resistant and 50 susceptible plants were selected from the F_2_ generation as two bulks for bulked segregate analysis (BSA) [29]. The bulked DNA samples were prepared by mixing an equal ratio of DNA extracted from 200 mg leaf samples using the cetyl trimethyl ammonium bromide (CTAB) method [30] with some modification [6]. These DNA samples were used for SLAF-seq analysis. DNA from the parents (CGN18423 and Moneymaker) and F_3_ lines was also prepared using the CTAB method for parental resequencing and marker testing, respectively.

Leaf samples of CGN18423 and Moneymaker were collected at 0, 4, 5, 7, 10, 13, 17 and 21 DAI for qRT-PCR analysis. Total RNA was extracted from leaf samples using a plant RNA mini kit (Watson, Beijing, China) according to the manufacturer’s handbook. First-strand cDNA was synthesized using an M-MLVRTase cDNA synthesis kit (Takara, Dalian, China) according to the manufacturer’s instructions.

### SLAF-seq and association analysis

A pre-design SLAF experiment was performed as described by [31]. According to the pre-design scheme, purified DNA was digested into DNA fragments of 364–464 bp in size, using an appropriate restriction enzyme (*Rsa*I). All subsequent SLAF-seq procedures were carried out referring to Sun et al. [19]. In the association analysis, P stands for the susceptible parent Moneymaker, M means the resistant parent CGN18423, aa represents the resistant pool and ab represents the susceptible pool. There are two genotypes for a single marker; aa1 and ab1 mean the depth of aa and ab samples with the first genotype, respectively, and aa2 and ab2 stand for the depth of aa and ab samples with the other genotype, respectively. These variables were used in the following calculations: aa index = aa1aa1/aa2; ab index = ab1ab1/ab2; ΔSNP index = aa index – ab index. The resistance phenotype is dominant, and the ratios of the two genotypes that can be detected are the same in unrelated markers, so the ΔSNP index of unrelated markers is equal to 0. Only one genotype of the ab sample can be detected in related markers; we stipulated that ab index equaled 0 or 1 and aa index had a value of 0.66 or 0.33, so the ΔSNP index of related markers was equal to −0.66 or 0.66, respectively. We calculated the ΔSNP index value of all markers and fitted these data; 95 % of the markers had fitting values greater than 0.3868831, so we chose this value to screen regions related to our target trait. Markers with fitting values higher than 0.3868831 were marked as Diff_Markers.

### Parental genome sequencing and data analysis

DNA from CGN18423 and Moneymaker was used in this part of analysis. All steps including sequencing, reads mapping, and analysis of SNP and insertion/deletion (InDel) polymorphisms were carried out according to Bai et al. [32]. The tomato genome sequence of Heinz 1706 [33] was used as the reference genome for reads mapping. SNPs in the association regions obtained from parental resequencing were analyzed in combination with those from F_2_ bulk SLAF-seq. SNPs that showed the Moneymaker base type in the susceptible pool, and both parental base types in the resistant pool were screened out to further narrow the mapping regions.

### Gene annotation and candidate gene screening

Genes in the final mapping regions were annotated according to the tomato genome annotations in the National Center for Biotechnology Information (NCBI) (http://www.ncbi.nlm.nih.gov/BLAST/) and SOL Genomics Network (SGN) (http://solgenomics.net/) websites. All genes with *Cf*-type characters in function (receptor-like) and structure (LRR domain) were selected as candidate genes.

### Quantitative real-time PCR analysis of candidate genes

qRT-PCR was carried out for all seven candidate genes. The primers (Table 4) for qRT-PCR were designed using the Primer 5.0 software. The qRT-PCR reaction was performed using an iQ5 system (Bio-Rad, USA). The reaction mixture contained 10 μL of 2× TransStart Top Green qPCR SuperMix (TransGen, China), 10 pM of each primer, 2 μL of cDNA templates (1:10 dilution), and sterile distilled water to make up a total volume of 20 μL. The thermal conditions (two-step method) were as of 95 °C for 10 s, T_m_ temperature for 30 s. To detect primer dimerization or other artifacts of amplification, a melting-curve analysis was performed immediately after completion of the RT-PCR (95 °C for 15 s and 55 °C for 15 s, followed by a slow increase of temperature by 0.5 °C per cycle to 95 °C with continuous measurement of fluorescence). The data were analyzed using the 2^–∆∆CT^ method [34] with *EFα1* as a reference gene for normalization [35].

**Table 4:** Primers used for qRT-PCR analysis and candidate loci sequencing

Primer name	Forward primer sequence (5’-3’)	Reverse primer sequence (5’-3’)
qRT-PCR primers		
qRT-5730A	CTGGATCGCCCATTTCACCT	CCCATGTAACTCTGTGCCTGA
qRT-5760A	TCTCATGGGTTACGGTTGTGG	TTCAAGTTTGTGTCTGATTTCTGT
qRT-5780A	CTCACCTCATTTGTGCCCCA	ATCACTTGCCCTGTCGTCTC
qRT-5870A	ATTGGCGGTGAAATCCCACA	AACCATAACCCACGAGCACC
qRT-6550A	TTTAGTGCAGAAATGGGTTGTGT	CACTTGTCCTGTCGTCTCGT
qRT-8390A	TCAGATGCTATTAGATTCAGACCTC	GTGGGAGACCTAACAGTGAGC
qRT-8410A	AGTTCTCAGTATCCAGAGTAGCCT	AGAAATTGGTCCTTCGAGATGGT
EFα1	CCACCAATCTTGTACACATCC	AGACCACCAAGTACTACTGCAC
Sequencing primers		
Cf-4/9-1	TTCGTAGACCAAATTAGCT	CTAGTTTGACCAATGTGAAG
Cf-4/9-2	TGGTCATTGATCTTCTAA	ACAGTTGGATTAGTAGGATTAC
Cf-4/9-3	AAGGTGTACCTTATAGCAT	GCGTTCGAGTGTTTTCGGGGAA
S5870A	AACAAAAGTCTTAATTATTTATA	ACCAATTTCCCAGTTCTCGATA
S5870B	GCTTATTCCAGTCCAATT	CACTTAATGCATTGGCAAAAAGA

### Candidate loci sequencing and DNA sequence analysis

To obtain detailed DNA sequence information, primers (Table 4) were designed for the candidate gene loci Solyc01g005870.1.1 and Solyc01g006550.2.1 using the Primer 5.0 software. The template sequences were from the reference genome sequence of SGN. The PCR products of CGN18423 and Moneymaker were purified with a PCR purification kit (Takara). The purified products were cloned into the pMD18-T vector (Takara) and sequenced. The DNA sequences we obtained were submitted to the NCBI database and analyzed using the Blast (http://blast.ncbi.nlm.nih.gov/Blast.cgi) and Open Reading Frame Finder (ORF Finder) (http://www.ncbi.nlm.nih.gov/gorf/gorf.html) tools of NCBI. Gene structure analysis was performed using the online tool SMART (http://smart.embl-heidelberg.de/). Other known *Cf* genes in the *Cf-4/9* locus (GenBank: AY639604.1, AJ002235.1, AJ002236.1) were also analyzed in combination with the candidate genes in this study to reveal evolutionary relationships.

### Marker developing and linkage analysis

Six markers including cleaved amplified polymorphic sequence (CAPS) markers and SCAR markers were designed based on sequence mutations in or near the candidate gene loci. Marker primers were designed using the Primer 5.0 software. The markers were first screened using CGN18423, Moneymaker and the F_1_ plants. Linkage analysis was then performed for these markers in an F_2_ population consisting of 345 plants. In this part, a special SCAR marker P7 was screened out. Therefore, three F_3_ lines (F_3_-1, derived from an F_2_ plant with the resistant homozygous genotype; F_3_-2, derived from an F_2_ plant with the resistant heterozygous genotype; F_3_-3, derived from an F_2_ plant with the susceptible homologous genotype; based on the P7 test), were used to further test the genotyping veracity of the marker P7.

### Availability of supporting data

The data sets supporting the results of this article are included within the article and its additional files.

## Additional file

Additional file 1: Table S1. Results from combined data analysis of parental resequencing and F_2_ SLAF-seq. Forty-three SNPs that showed the Moneymaker base type in the susceptible pool, and both parental base types in the resistant pool were screened out. Of the 43 SNPs, 34 were distributed in an approximately 2.14-Mb region in association region I, one was in association region II and eight were in association region III. Seven *Cf*-type genes were identified in association region I. (DOCX 16 kb)

## Abbreviations

*Avr*: Avirulence gene
BSA: bulked segregate analysis
*C. fulvum*: *Cladosporium fulvum*
CAPS: cleaved amplified polymorphic sequences
CTAB: CetyI TrimethyI Ammonium Bromide
DAI: days after inoculation
*Hcr2*: homologues of *Cladosporium* resistance gene *Cf-2*
*Hcr9*: homologues of *Cladosporium* resistance gene *Cf-9*
HR: hypersensitive response
InDel: insertion/deletion
LRR: leucine-rich repeat
MAS: marker assistant selection
NCBI: National Center for Biotechnology Information
ORF: open reading frame finder
qRT-PCR: quantitative real-time PCR
QTL: quantitative trait loci
*R* gene: resistance gene
SCAR: sequence characterized amplified region
SGN: SOL Genomics Network
SLAF-seq: specific-locus amplified fragment sequencing
SNP: single nucleotide polymorphism
VIGS: virus induced gene silencing
