# Supplementary material for: Mapping and candidate gene screening of tomato Cladosporium fulvum-resistant gene Cf-19, based on high-throughput sequencing technology
Source: BMC Plant Biol. 2016 Feb 25;16:51. doi: 10.1186/s12870-016-0737-0 (PMC4766677; doi:10.1186/s12870-016-0737-0)
Supplement: Additional file 1: Table S1. — Results from combined data analysis of parental resequencing and F2 SLAF-seq. Forty-three SNPs that showed the Moneymaker base type in the susceptible pool, and both parental base types in the resistant pool were screened out. Of the 43 SNPs, 34 were distributed in an approximately 2.14-Mb region in association region I, one was in association region II and eight were in association region III. Seven Cf-type genes were identified in association region I. (DOCX 16 kb) [file 12870_2016_737_MOESM1_ESM.docx]

**Table S1** Results from combined data analysis of parental resequencing and F_2_ SLAF-seq

| Assoc-iation region | Diff-Marker name | genome positon | Base type | | Base type | | Number of resistance- type gene |
| --- | --- | --- | --- | --- | --- | --- | --- |
|  |  |  | CGN1-8423 | Money-maker | Resistant pool | Susceptib-le pool |  |
| I | Marker276940 | 429823 | A | G | A/G | G | 7 |
|  | Marker244763 | 665377 | T | A | T/A | A |  |
|  | Marker287336 | 848605 | A | G | A/G | G |  |
|  | Marker276311 | 951133 | G | A | G/A | A |  |
|  | Marker259248 | 1132037 | A | G | A/G | G |  |
|  | Marker259248 | 1132072 | C | A | C/A | A |  |
|  | Marker238438 | 1155703 | C | T | C/T | T |  |
|  | Marker260370 | 1165016 | A | G | A/G | G |  |
|  | Marker257689 | 1363170 | G | A | G/A | A |  |
|  | Marker239897 | 1452608 | T | G | T/G | G |  |
|  | Marker239897 | 1452620 | A | G | A/G | G |  |
|  | Marker273470 | 1545177 | A | C | A/C | C |  |
|  | Marker249650 | 1632525 | G | A | G/A | A |  |
|  | Marker249650 | 1632533 | T | A | T/A | A |  |
|  | Marker249650 | 1632571 | C | T | C/T | T |  |
|  | Marker258874 | 1711997 | A | C | A/C | C |  |
|  | Marker249868 | 1722379 | G | T | G/T | T |  |
|  | Marker249868 | 1722390 | T | C | T/C | C |  |
|  | Marker237475 | 1741687 | A | G | A/G | G |  |
|  | Marker276087 | 1765383 | G | A | G/A | A |  |
|  | Marker279981 | 1811696 | T | A | T/A | A |  |
|  | Marker277549 | 1915350 | G | A | G/A | A |  |
|  | Marker240775 | 1929701 | A | G | A/G | G |  |
|  | Marker275731 | 1946574 | T | A | T/A | A |  |
|  | Marker261212 | 2026330 | T | C | T/C | C |  |
|  | Marker287895 | 2086323 | T | C | T/C | C |  |
|  | Marker265576 | 2092612 | G | A | G/A | A |  |
|  | Marker283060 | 2110802 | C | T | C/T | T |  |
|  | Marker258537 | 2237808 | T | A | T/A | A |  |
|  | Marker240258 | 2392118 | A | G | A/G | G |  |
|  | Marker251652 | 2424224 | A | G | A/G | G |  |
|  | Marker247736 | 2425613 | C | T | C/T | T |  |
|  | Marker249334 | 2430647 | T | C | T/C | C |  |
|  | Marker251999 | 2570961 | T | G | T/G | G |  |
| II | Marker238368 | 20671061 | C | A | C/A | A | 0 |
| III | Marker265413 | 44548556 | A | C | A/C | C | 0 |
|  | Marker276909 | 44868373 | T | A | T/A | A |  |
|  | Marker283801 | 44938283 | T | C | T/C | C |  |
|  | Marker287188 | 45500452 | T | G | T/G | G |  |
|  | Marker266955 | 47269989 | C | G | C/G | G |  |
|  | Marker248323 | 47935282 | A | G | A/G | G |  |
|  | Marker288706 | 48140421 | A | C | A/C | C |  |
|  | Marker244693 | 48987555 | A | C | A/C | C |  |
